# Private water distribution as a potential everyday risk: The case of Goba, Dar es Salaam

**DOI:** 10.4102/jamba.v11i1.775

**Published:** 2019-08-21

**Authors:** Tumpale Sakijege

**Affiliations:** 1 Department of Urban and Regional Planning, School of Spatial Planning and Social Sciences, Ardhi University, Dar es Salaam, United Republic of Tanzania

**Keywords:** peri-urban, groundwater and sanitation issues, risk, Goba, Dar es Salaam

## Abstract

A large number of peri-urban settlements in developing countries, including Goba in Tanzania, fall short of government supplied water. The inability of the Government to budget and prioritise its budget poses a serious problem to meet the water demand, a few residents in peri-urban settlements use other sources of water, including groundwater. However, the quality and suitability safety of such groundwater are questionable. This research of Goba settlement was undertaken to explore the reality of what happens and how problems can be resolved. The research methodology deployed in-depth interviews, physical observations, photographing and mapping and analysing and testing various water samples in a laboratory. From the study several conclusions could be drawn: most of the current laws relating to groundwater and sanitation were violated, the distribution of water to neighbours compounded problems. The major diseases typhoid, diarrhoea and dysentery were common. People themselves can take the necessary precaution by the choice of locating waste water so that it does not contaminate, boiling drinking water, etc. The devolution of the power from the Central Government and Ministries to District and down to the wards would go a long way to bring public/private partnership to be meaningful. The evidence from Goba points to the prevailing situation and what could be done to bring improvements.

## Introduction

The Government of Tanzania made a renewed and massive support for water and sanitation throughout the country following the Third United Nations (UN) Water Development Decade. Massive donor assistance in the late 1970s actually considered the practical steps as a priority. This was a stark contrast to the urban bias during the pre- and post-colonial situation. Fresh water used to be piped by two very large schemes, via the Lower and Upper Ruvu water schemes (see numerous reports Bureau of Resources Assessment and Land Use Planning/Institute of Resource Assessment, BRALUP/IRA).

Access to safe and clean water was recognised as a human right by the United Nations (UN) General Assembly on 28 July 2010 (WHO 2016). Retrospectively, this means that every person is entitled to get access to safe, clean and affordable water. Similarly, water is essential for promoting inclusive sustainable development (UNDP 2016). Despite such aspiration, safe water access has remained a critical challenge over the years (Allen et al., 2017).

When faced persistent problems of water shortage, many people in all countries are forced to use any unsafe sources of water for domestic purposes (Teresa 2014). Some of these sources include groundwater, rainwater and various types of surface-water sources, such as rivers, lakes, ponds, creeks, irrigation channels, seawater and constructed reservoirs (WHO 2016). This in turn leads to a number of risks (Echazú et al. 2015), leading to waterborne diseases that cause numerous deaths with children and vulnerable groups being affected more (WHO 2016). The United Nations Educational, Scientific and Cultural Organization (UNESCO) concluded that:

… Water quality deterioration is projected to rapidly increase over the next several decades which, in turn, will increase risks to human health, economic development and ecosystems … (Veolia & IFPRI 2015:3 as cited in WWAP 2016:20)

Moreover, the United Nations Sustainable Development Goal 6 emphasises on the need to ‘Ensure availability and sustainable management of water and sanitation for all’ (UN, 2016:6). One of the indicators that are needed to monitor this goal is ‘To achieve universal and equitable access to safe and affordable drinking water by 2030’ (UN 2016). Notably, about 1.8 billion people use a source of water that is fecally contaminated and more than 2 million people worldwide die every year from diarrhoeal diseases (CRO-Forum 2016). Likewise, a number of people in developing countries have been left behind. For instance, more than 40 per cent of the people around the world are affected by water scarcity. (UNDP 2016:8)

In Africa, access to reliable and affordable water has been a critical issue because the population increases rapidly compared with the capacity of governments to provide water services (Kombe, Ndezi & Hofmann 2015). Africa, as one of the rapidly urbanising continents, has a projected population of 2 billion by 2050 (AfDB, OECD & UNDP 2015). This rapid increase in Africa’s population leads to increased demand for basic services particularly water. In fact, Africa is considered a scary story depicting vulnerability to increasing water stress, among others (WWAP 2016). As a result, competition for water will intensify among users.

Demand for water in Africa has intensified with the high increase in population. Among 800 million people who lack access to safe and clean water in the world, 344 million (equivalent to 43%) reside in sub-Saharan Africa (Adams & Zulu 2015; UNICEF & WHO 2015). Besides over-centralisation, there are also several reasons that have led to poor water supply, these include failure of the privatisation (Ahlers, Schwartz & Guida 2013), poor management (Kombe et al.2015), inability of governments to provide water services to cope with rapid increase of population (Andreasen & Møller-Jensen 2016) and limited public investment in water infrastructure (Liddle et al. 2016). Based on the fact that water has no substitute (UNICEF/WHO 2012), people will use whatever source of water without even considering its quality.

In Tanzania, the responsibility of supplying safe and clean water rests with the Ministry, of Water and Irrigation. Government, through the ministry, has failed to ensure that each and every citizen get access to safe and clean water. As a result, a number of private water vendors have emerged in areas that experience water scarcity. For instance, 70% of the population in the low-income areas rely on the informal service provider (Gronemeier & Moore 2014). In line with this, a number of research studies (Nganyanyuka et al. 2014; Kombe et al. 2015; Allen et al. 2017; Mapunda, Chen & Yu 2018) acknowledge the importance of private water supplier in reducing water shortage. Unfortunately, these researchers examined the positive side of the private water supply and thus leaving issues related to water quality, water use permit and the related risks unexplored. It is high time that all these issues are well explored, and solutions sought. Hence, this article set out to bridge this glaring gap by providing more insights into procedures people use to extract groundwater and identify groups that were most vulnerable to water-related risks. More specifically, the article examined the procedure followed to allow individuals drill and distribute water. It also investigated the way water quality assurance and the related routine water quality tests were being done immediately after extraction.

### Literature review

The foregoing section has summarised the research issue and its manifestation from the global level. Conversely, the sections below describe the magnitude of the situation in the context of Dar es Salaam.

#### Status quo in Dar es Salaam

Dar es Salaam Water and Sanitation Authority (DAWASA) and Dar es Salaam Water and Sanitation Corporation (DAWASCO) are the two public entities that manage the city’s water supply and sanitation system. The former is the owner of the assets for water supply and sewerage services in Dar es Salaam region and part of the Kibaha and Bagamoyo districts and it is responsible for planning, procurement and implementation of strategic capital works. On the contrary, the latter is responsible for providing water supply and sewerage services in the DAWASA designated area through a lease contract (EWURA 2017). The primary agency authorised to carry out water and waste water quality monitoring to DAWASA and DAWASCO is Energy and Water Utilities Regulatory Authority (EWURA). The aim has essentially been to establish whether water supplied complies with Tanzania Bureau of Standards (TBS) requirements and gives directives for remedial measures if there are anomalies (EWURA 2017).

As of 2012, 51% of the Dar es Salaam population was estimated to get water directly from the official public water supplier (EWURA 2012). This implies that 49% who are the majority live in informal settlement and have to use alternative practices to meet their water access and needs. The quality issue is often considerably ignored compared with the public water supplies whose quality is controlled by EWURA. Precisely, it states:

Section 11(3) of the *Water Resources Management Act, 2009*, states that:

… Any person being the legal owner or occupier of any land may construct a shallow hand-dug well and use the water for domestic purposes without a Groundwater Permit issued under this Act subject to any limitation on the depth of such wells in any area as may be prescribed in Regulations by the Minister. (United Republic of Tanzania [URT] 2009:366)

This section provides for an application of groundwater use permit when one intends to use groundwater for the use other than domestic and thus obliged to apply for water use permit (an additional Section 75 states). Exploring whether private water supplier in peri-urban areas obtain water use permit prior to distribution to their neighbours is crucial as it will provide a holistic understanding of how the informal water supply system contributes to increasing water supply in peri-urban areas without compromising consumers’ health.

#### The neglect of everyday risks

The level of understanding of risks associated with the use of water supplied by private proprietors varies considerably. The broad observations are twofold: there is limited understanding of risks as a result of using water supplied by private proprietors; there is noticeable neglect of quality of water supplied by private proprietors regardless of their importance in increasing water supply. These annotations hold true in Tanzania, where studies on the risks and access to water have been conducted. The few studies that have been conducted on the access to water have tended to concentrate on citizens’ strategies for accessing water (Kombe et al. 2015) and the role of informal small-scale water supply system in resolving drinking water shortages (Mapunda et al. 2018). With regard to risks, previous studies that were conducted in Tanzania focused on flood associated risks (Sakijege, Lupala & Sheuya 2012), urban fire risks (Kachenje, Kihila & Nguluma 2010), fire disaster preparedness in higher learning institutions (Kihila 2017) and ineffective urban planning practices to risks accumulation in urban areas (Malele 2009).

So far, there are no traceable studies examining risks because of the use of groundwater. Almost half of the population in Dar es Salaam and Tanzania in general is estimated to use groundwater for home consumption (EWURA 2012).

Numerous studies conducted in the field of disaster in general (Jha, Bloch & Lamond 2012; Fatti & Patel 2013; Centre for Research on the Epidemiology of Disasters [CRED] and UN-Disaster Risk Reduction 2015; EM-DAT 2016) support the arguments that disasters lead to death, leave many people homeless and cause damage to infrastructure worth billions of dollars. However, previous researchers have tended to neglect small risks that occur in society everyday whose impacts take time to be noticed. It is worthwhile to note that no human being who has never used water; it is therefore necessary for humans’ survival. Risk resulting from the use of contaminated water is extremely difficult to determine until occurrence of great eruption of diseases to the extent of causing even death. The largest percentage of developing countries deals with disasters after they have occurred (Sakijege et al. 2012) and conveniently forgotten until the next disaster.

#### Water quality and water-related risks

Groundwater has been termed the ‘hidden sea’ – it is hidden because it is not visible (Chapelle & Chapelle 1997). This highlights that particular attention is needed to ascertain the quality of groundwater before home consumption (WHO 2006). Nonetheless, outbreaks of disease from contaminated groundwater sources are reported from countries at all levels of economic development (WHO 2006). As population pressures increase in many parts of the world, it is becoming increasingly apparent that the issues of water quality waterborne infectious risks cannot be ignored.

### Conceptual framework

This study emphasises that in order to minimise possible risks associated with contaminated water, there is a need for private proprietors to adhere to professional procedures when extracting and distributing groundwater. While a large number of residents in Dar es Salaam are not served with government-piped water, nevertheless these people need water. Poor people are forced to use readily available water sources closer to their residences. The irony is that such water cost much more than the one enjoyed by residents who have privilege to access government-piped water in the elite areas of Oysterbay, Masaki and Upanga. In most peri-urban parts of the city of Dar es Salaam (Mbezi, Kijichi, Goba, Kitunda, Salasala, Tegeta and Kinyerezi) there are a number of individuals with private bore holes and deep wells distributing water to neighbours at a cost, which is often considerably higher compared with the official rates charged by DAWASCO (Kombe et al. 2015; Pauschert, Gronemeier & Jebens 2012).

Groundwater is vulnerable because they are contaminated by a number of human activities (WHO 1997). Some of the human activities include waste dumps (Abelson 1990), sanitary facilities (Langergraber & Muellegger 2005) and untreated industrial effluents (Mondal, Saxena & Singh 2005). Further to that, geology and topography of the area must be considered because they determine infiltration rate (WHO 1997). Given these factors, expert advice should be sought when drilling boreholes (WHO 1997). Furthermore, WHO (1997) provides that a borehole should be placed at minimum safe distance (MSD) of between 50 m and 100 m from the potentially polluting activities like sanitary facilities.

Another issue that is of paramount importance when extracting groundwater is obtaining a permit. Permit enables authorities to ensure wells are properly constructed, repaired, so that water quality and public health are protected. It should be noted that water use permit and substantiating water quality is one of the prerequisites for water supply services (Sima & Elimelech 2011; URT 2009). In areas where population density is high and human use of the land is intensive, groundwater is especially vulnerable (Pawari & Gawande 2015). This means that increasing housing densification contributes to risk of groundwater contamination. Unless managed effectively cleanness and safety of groundwater need to be verified especially in areas with concentration of buildings (WHO 2016). This article, therefore, examined issues of expert involvement, water quality test, water use permit and MSD when extracting and distributing groundwater.

## Research method and design

### Setting

Six criteria were developed in order to identify and select a case study that was suitable for conducting the study; they were (1) a settlement located in peri-urban areas of Dar es Salaam, (2) existence of formal and informal housing development, (3) a settlement that the majority of residents lack government supplied water, (4) residents who demonstrated effective responses for containing water shortage problem, (5) a settlement traversed by a major highway and thus attract a number of people, (6) a settlement which needed critical intervention in terms of risk reduction measures. All informal settlements in Dar es Salaam were subjected to evaluation based on the aforementioned criteria and Goba sub-ward was chosen for an in-depth case study.

### Population and sampling procedures

This study used both qualitative and quantitative methods of assessment. The qualitative methods of assessment consisted of in-depth interviews with selected respondents, physical observations, photographing and mapping. Household interviews were held with borehole owners to identify procedures used to extract groundwater and distribute it to neighbours. The interviews were also meant to establish whether or not water quality test is normally done. Moreover, the study interviewed households that receive water service from boreholes/deep-well owners for the purpose of capturing their perceptions on the service they receive and possible risks facing them. Furthermore, quotes from interviews were used to enhance clarifications. Quantitative assessment was used for taking measurements of distance from well to sanitary facilities. Further to that, in order to substantiate risks (water pollution issue) samples of water were taken from three boreholes for laboratory tests.

The sampling techniques employed in this study included both probability and non-probability sampling. On one hand, purposive sampling design which forms part of non-probabilistic design was employed because it power lies in selecting information-richest cases for in-depth studies (Kothari 2004). This argument was considered valid in this research, the research aimed at investigating procedures used to extract and distribute groundwater. Within the case study area, the researcher expected that not all households in the entire settlement owned bore holes. That being the case; households with private boreholes were considered potential case studies in accomplishing the research goal.

Goba (with 1185 households) sub-ward has a total of 17 households with private boreholes. Considering time and cost, it was impractical to interview all borehole owners. This necessitated the selection of a sample size. Kothari (2004) argues that in purposive sampling design, there is no assurance that every element has some specifiable chance of being included. Thus, it was important to apply random sampling within purposive sampling in order to come up with a representative sample with no bias. Thus, simple random sampling technique was applied to select household for interview. A simple random sampling was meant to be an unbiased representation of a group. Using lottery method, 10 owners of boreholes were chosen out of a hat from a group of 17 borehole owners.

In order to get people that are connected by water, it was crucial to employ snowball sampling technique. This is a chain referral process that falls under non-probabilistic sampling techniques which allow the researcher to reach populations that are difficult to sample. In the first place, every private borehole owner interviewed was asked to identify any person that was receiving water service from his/her source. The identified household was the first one to be interviewed and asked to refer others who were receiving similar service. The interview stopped after the researcher was satisfied that there was no new information. About 32 respondents were interviewed.

### Case study area and its main characteristics

**FIGURE 1:**
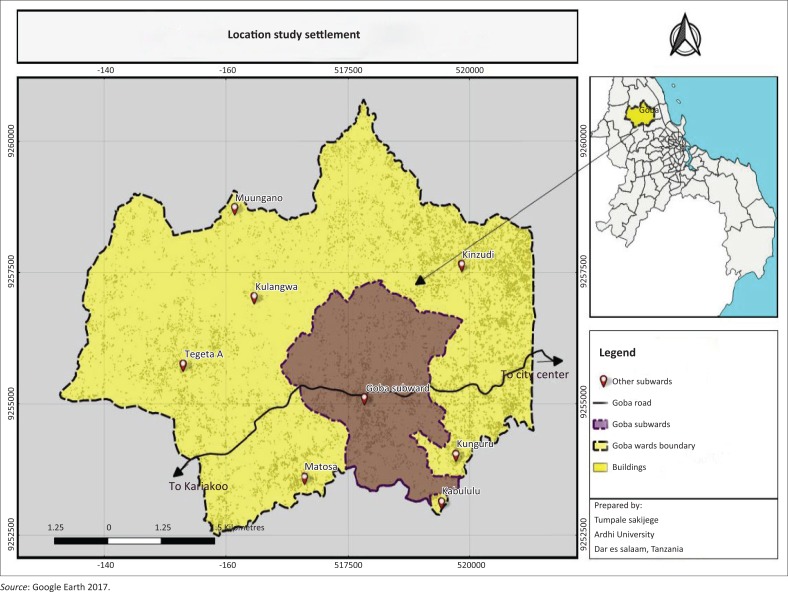
Location of study settlement.

Dar es Salaam region is composed of five municipalities, namely Kinondoni, Ubungo, Ilala, Kigamboni and Temeke. Each municipality is autonomous, but administratively the five are coordinated by the Dar es Salaam City Council. The study area of Goba is located in Ubungo Municipality. It covers an area of 4719.48 hectares (ha) with 42 669 inhabitants Goba sub-ward which is the study settlement is one of the sub-wards within Goba Ward others being Kibululu, Matosa, Kunguru, Kinzudi, Tegeta A, Kulangwa and Muungano (Figure 1). Geographically, Goba sub-ward is about 30 km from Dar es Salaam City centre. It stretches along Goba Road, which is a trunk road that leads to Bagamoyo road on the eastern side and Morogoro road on the western side. Being far from the city centre, it makes the area attractive for settlement and its associated activities especially for those who dislike chaos of areas around the city centre. Goba sub-ward has the population of 5213 people (Table 1).

**TABLE 1:** Household size and number of people.

S. No.	Sub-ward name	Number of household	Number of people
1	Kinzudi	1178	7013
2	Kunguru	1186	6097
3	Kibululu	1594	5181
4	Tegeta ‘A’	1566	6892
5	Goba	1185	5213
6	Muungano	1181	5214
7	Matosa	1386	6098
8	Kulangwa	1197	3268
**Total**	**-**	**10 473**	**44 976**

*Source*: United Republic of Tanzania (URT), 2013, *Population and housing census*, Population distribution by administrative areas, Government Printers, Dar es Salaam, Tanzania and Field work on 24.05.2018, Goba Sub-Ward Office

The settlement is characterised by a mixture of hilly topography and gentle slope, in addition it has partly been developed formally but large part is informally developed. The major reason of attraction to this settlement is its strategic location with better outlets to all municipalities including the city centre and the regionally famous shopping centre of Kariakoo.

### Ethical considerations

The data for this article were obtained from a study conducted at Goba from March to June 2018. The proposal was reviewed at the university and submitted for funding (without success). Later, approval to commence the study was granted.

## Findings of the study

**FIGURE 2:**
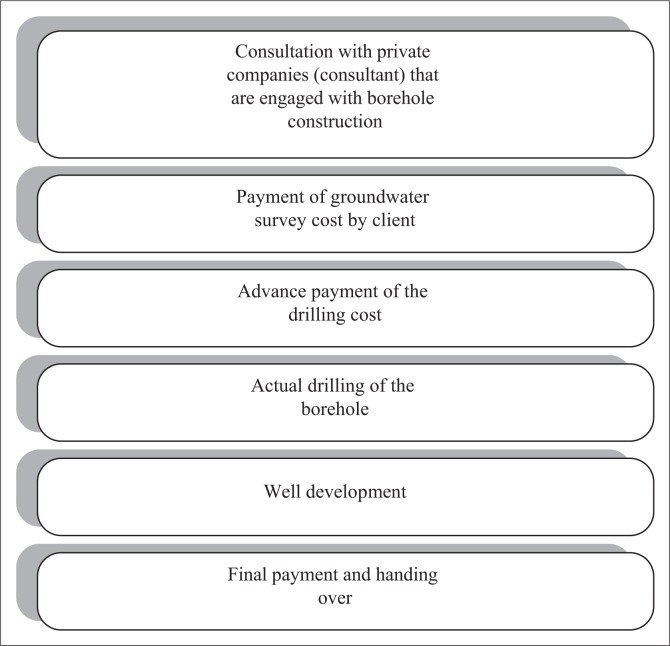
Borehole construction procedures.

The results indicated that private proprietors tend to engage private companies in an effort to extract groundwater. Out of 10 borehole owners interviewed, six (60%) reported to engage private companies for extracting groundwater. The procedures used are summarised in Figure 2. However, as it can be seen in Figure 2, the issue of water quality test prior to the use is not reflected. The remaining four respondents (40%) admitted to engage Drill and Dam Construction Agency (DDCA) in extracting groundwater. When asked to show the laboratory results, none of these 40% who claimed to get service from DDCA was able to provide water quality test results. This presented critical questions on the reliability of the information given. Where water quality analysis cannot be performed, it is very difficult to establish what needs to be done to protect the water source and minimise water-related risks (WHO 2006).

As per the *Tanzania Water Resources Management Act*, any person should request for a water use permit prior to distribution of groundwater to neighbours. Based on the study conducted in Goba, 9 (equivalent to 90%) out of 10 owners of boreholes sells drilled groundwater. Surprisingly, when asked about the permit for the use of water for businesses they did not have such permits. The water-related laws explicitly emphasised the importance of water quality test prior to the issuance of water use permit; however, this is not reflected on the ground. The practice of distributing water without permit has to do with government laxity in enforcing existing laws, whereas nothing is being done so far to curb the situation.

Most deep wells are built within residential plots. The study revealed that within residential areas, there are sanitary facilities which have been built without considering MSD to the wells. The distance between the wells and septic tanks for example ranges from 8 m to 12 m, whereas the WHO standards suggest the range of 50 m – 100 m depending on the type of geology. Approximately 60% of the borehole owners interviewed admitted to have not been paying attention to the required MSD between septic tanks and boreholes because at first when constructing houses, they believed that they would get water through the government system.

Water supply to neighbours is carried out for two main purposes (1) helping colleague to reduce the problem of water accessibility and (2) one of the income-generating strategies. There are those who pay connection costs directly to the deep-well owners (47%) and the deep-well owners take responsibility of plumbing work. There are those who are asked to do the plumbing work by themselves (41.8%). The rest (11.2%) carry water on their heads or through wheelbarrows at a price of 200 per bucket. In average borehole owners have between 25 and 50 customers. The cost of one unit of water (1000 L) ranges from (Tanzanian shilling) TZS3000.00 to TZS5000.00. The majority of interviewed customers (82.4%) were dissatisfied with the salinity in water (although they acknowledged that water from private boreholes is very helpful given the absence of government supplied water) and the high price charged compared with those who are privileged to get government supplied water (1700/1000 L).

**FIGURE 3:**
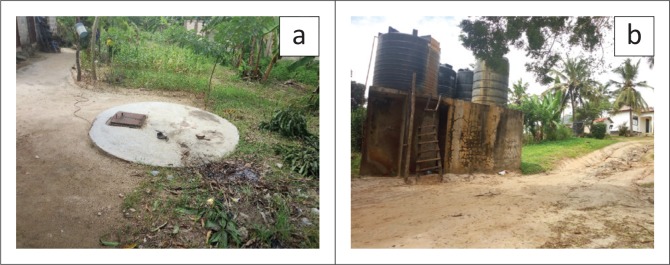
(a) Storage tank on the ground surface, (b) Overhead storage tank much safer than ground storage tanks which are risky.

Water stored in tanks was found to be available, every owner of the deep-well store water in storage tanks before selling to customers. This reduces complaints about shortage of water from customers because it ensures its constant supply. However, when asked how often they clean storage tanks, 52.9% of the respondents claimed to do it once a year. This practice can increase everyday water-related risks especially by considering that some of the storage tanks are constructed on the ground at the surface level and hence vulnerable because contaminants can enter the tanks through the lid of the storage facility (Figure 3). Potential contaminants can be found in every area and thus they can easily enter storage facility that fitted on the ground surface when it rains.

**FIGURE 4:**
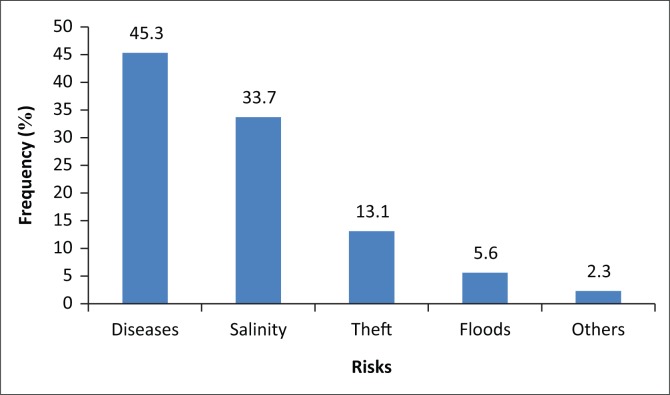
Risks facing Goba sub-ward residents.

When asked to state the most serious risk that facing them, disease ranked highest (Figure 4), 45.3% of the respondents reported that diseases where the most serious risks in Goba generally, whereas 33.7% felt that salt water is also a risk especially when it comes to drinking and washing clothes.

‘It’s too hard to adapt to the use of salty water, but no way because government supplied water are very expensive.’ (Participant 7, male, economist, adult, graduate)

‘I always use salt water for washing clothes, cleaning, gardening, when it comes to drinking and cooking I prefer buying from trucks at a price of 15 000 per 1000 L.’ (Participant 3, female, entrepreneur, adult, secondary education)

It was observed that a household’s water source may also contribute to different kind of diseases. Table 2 presents the type of diseases that affects residents in the Goba sub-ward. Majorities are affected by typhoid (22.3%), and others are affected by diarrhoea (18.4%), dysentery (16.3) and other diseases (43%). In areas like the Goba Ward where majority uses water from private suppliers, incidence of various waterborne diseases as they have been reported by respondents is greatly influenced by the quality of water they use.

**TABLE 2:** Most commonly reported water-related diseases.

S. No.	Diseases	% of respondents
1	Typhoid	22.3
2	Diarrhoea	18.4
3	Dysentery	16.3
4	Others diseases	43

**FIGURE 5:**
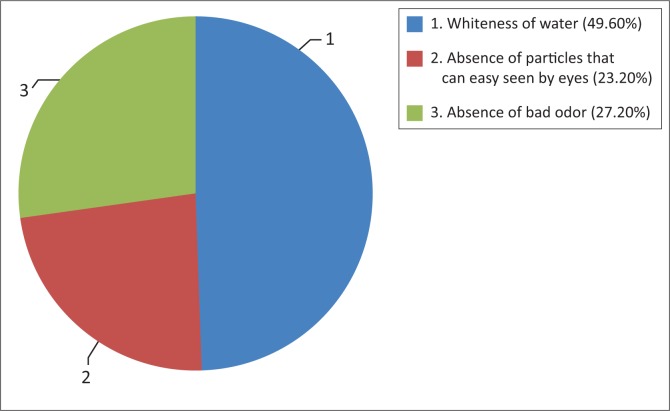
Perception on the water quality.

When asked about the quality of water they use, majority (68.7%) said that water they are using is safe and clean because it is from the ground source where they believe is free from contamination. It was indeed more crucial to understand the way they perceive ‘clean and safe water’ (Figure 5).

The major problem raised by the people who use groundwater at Goba was the salinity in water noticed by people when using water for drinking and washing clothes. Nevertheless, water users at Goba believe that water they use is clean and safe for use; however, results show that water users have not stopped treating water through different methods such as boiling and the use of waterguard.

**FIGURE 6:**
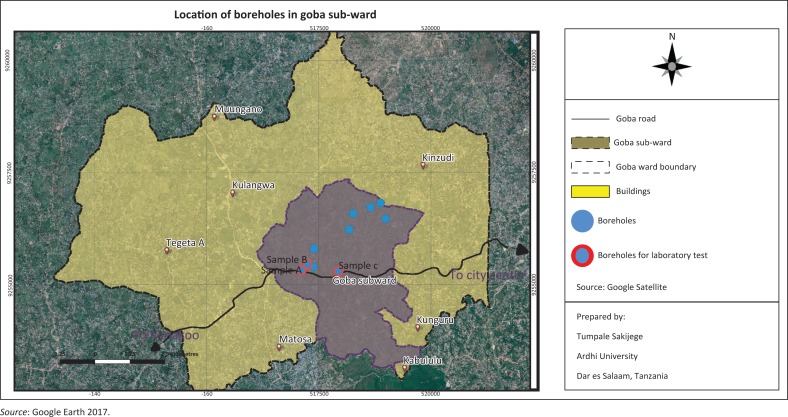
Location of boreholes in Goba sub-ward.

The study also picked up sample of water from three different wells to examine the extent of water contamination. Table 3 shows results of water tests and Figure 6 shows location of wells where water samples were taken. Analysis of laboratory results revealed that ‘A’ does not show any water contamination and the salinity (electric conductivity [EC] 1909 *µ*S/cm) is fairly good compared with Tanzanian standards (2000 *µ*S/cm). It should be noted that electrical conductivity is used routinely to measure salinity (Cañedo-Argüelles et al. 2013). The situation is not satisfactory in samples ‘B’ and ‘C’ where sample B has shown 16 count/100 mL and 29 count/100 mL for Faecal coliform and Total coliform, respectively, whereas the standard is zero count/100 mL as per Tanzanian standards and as per World Health Organization’s drinking water quality specification of 0 colonies/100 mL. Faecal coliform and Total coliform for sample C have shown 7 count/100 mL and 13 count/100 mL correspondingly. Moreover, results from sample ‘C’ show EC of 2400 *µ*S/cm, the figure which is above normal and thus depicts high salinity level (Cañedo-Argüelles et al. 2013). Several health issues originate from high intake of salt and drinking contaminated water. For example, high blood pressure (hypertension) (Khan et al. 2011) and heart diseases (Mohammad 2016). Dysentery, typhoid and diarrhoea are among the diseases that have been reported to be caused by drinking contaminated water, certainly according to interview residents in Goba sub-ward are affected by similar diseases. Among Typhoid has a high percentage (22.3%) followed by diarrhoea (18.4%) and dysentery (16.3%). Given the extent of Faecal coliform and Total coliform in the samples ‘B’ and ‘C’, and given presence of water-related diseases as reported by respondents further research is urgently required to determine potential risk of water pollution on all private deep wells, therefore, promote appropriate prevention strategies.

**TABLE 3:** Laboratory results of water test.

S.No.	Parameter	Units	A	B	C	TZS:789:2003 STANDARDS	Remarks
5	Electric conductivity (measures salinity)	*µ*S/cm	1909	1791	2400	2000	In conformity with Tanzania and WHO standards except Sample ‘C’
8	Faecal coliform	Count/100 mL	0	16	7	0	Risks in ‘B’ and ‘C’ samples
9	Total coliform	Count/100 mL	0	29	13	0	Risks in ‘B’ and ‘C’ samples

*Source*: Environmental Engineering Laboratory, Ardhi University, May 2018.

TZS 789:2003 – Drinking (potable) water – Specification (TBS 2003).

The majority of borehole owners are not visited or inspected by TBS or any authority responsible for controlling water quality. A TBS representative could not say whether they are responsible for controlling water quality in areas like Goba where majority depends on groundwater.

## Discussion

Individual efforts to extract ground water and sell to others are useful intervention in increasing water supply especially in peri-urban areas that lack public water. These findings support the fact that governments in developing countries failed to provide water services to cope with rapid increase of population (Kombe et al. 2015; Andreasen & Møller-Jensen 2016).

The study has shown that water from boreholes is preferred by residents in Goba sub-ward because they lack an alternative. Water from boreholes is acceptable as long as professional procedures and health issue are considered. However, more than half of the respondents do not follow water testing procedures before selling it to others. Distributing water without testing water quality is endangering consumers’ health and thus aggravating water-related risks (Mandour 2012).

Diseases are the major risks that have been reported to affect residents in Goba, in fact most of them are water-related diseases (dysentery, typhoid and diarrhoea), this suggests possibility of existence of water contamination, and thus majority of residents that depend on groundwater are at risk. Typhoid and diarrhoea are the most common conditions attributable to waterborne pathogens linked with water salinity (Vineis, Chan & Khan 2011). Furthermore, laboratory results, which indicated presence of coliform bacteria, are an indication that groundwater are contaminated and possibly diseases that have been reported by respondents are caused by drinking contaminated water and thus extra care is needed. Water pollution caused by faecal contamination is a serious problem because of the potential for contracting diseases from pathogens (LeChevallier, Welch & Smith et al. 1996), thus majority that uses groundwater may be at great risks should the current trend continue.

The study revealed that boreholes were constructed at a MSD below WHO standards. This is not acceptable as they can easily get contamination and causes risk to users because human activities are major source of pollution among others.

Although majority of those interviewed claim to engage experts when extracting borehole, the study revealed that experts are used to explore suitable area for extracting borehole without considering potentially polluting activities. That is why MSD as recommended by WHO is not adhered. Should the current trend continue, individual’s effort can change and be a major source of disasters given significant increase of population in urban areas.

Energy and Water Utilities Regulatory Authority as an authority for water quality monitoring has been assigned by law to monitor only DAWASA and DAWASCO. Similarly, the TBS as an authority for formulation of standards, metrology quality control, testing and calibration does not seem involved in monitoring and controlling quality of water. Private borehole owners have been left to professionally construct such facilities and monitor the quality of water. All these three responsible authorities ignore citizens’ initiatives of maximising water supply in areas where themselves proven incapable.

However, this report supports the argument that water risks are often underestimated, disregarded or simply ignored consequently, they result in a global lack of preparation for future impacts (CRO-Forum 2016). It is of course a common practice in most countries where they concentrate in fortifying strategic political or economic sectors and areas (Mitlin & Satterthwaite 2013). Although the reported risks in Goba are relatively small, they will accumulate and lead to a major disaster (eruption of waterborne diseases) should the current trend continue.

## Conclusion

This study focused attention on the number of issues, all of which aimed at improving the understanding of the risks facing informal settlements dwellers in peri-urban areas following the use of groundwater supplied by private households. The study draws the following conclusions:

First and foremost, the study has established a notable increase in the number of policies that are being ironically not implemented. Equally importantly, water-related diseases were the major risks reported, apparently resulting from consumption of contaminated water. Based on the results, the key issues are hereunder highlighted:

- Failure to follow groundwater extraction and water testing procedures before distributing water to others
- Low-literacy rate and understanding of the potential risks resulted from the use of groundwater
- The laxity of the government institutions in implementing laws

In summary, the results from this study serve as a litmus indication pointing to how policy makers can harness private/public partnerships. It is also worth underscoring that water and disaster management policies should focus on monitoring water extraction and distribution in areas where the government supplied water is lacking. The general tone running throughout the study strongly emphasises the ideal that the general public would like to have an improved water supply system because this should be the first and foremost priority.
